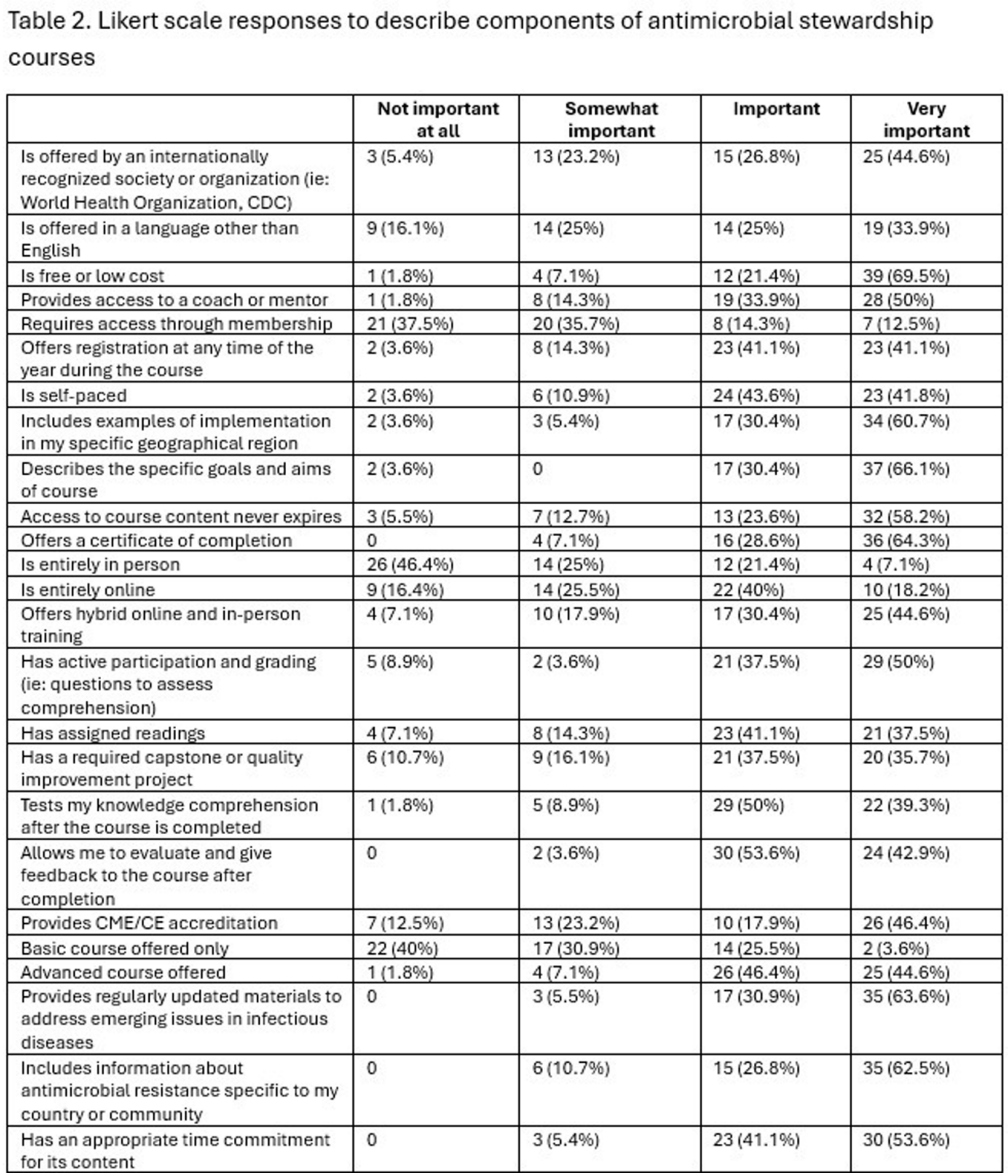# 220 Inpatient Urine Culture Yield Was Not Higher with Fever, Denver Safety-Net Health System, April 2016–December 2025

**DOI:** 10.1017/ash.2026.10444

**Published:** 2026-06-23

**Authors:** Anita Shallal, Gina Maki, Tyler Prentiss, Alle Taylor, Marcus Zervos, Twisha Patel

**Affiliations:** 1 Henry Ford Hospital; 2 Henry Ford Health System; 3 Henry Ford Health System, Detroit-USA; 4 Centers for Disease Control and Prevention

## Abstract

**Background:** Previously published data demonstrate the need to improve education and training of healthcare workers in antimicrobial stewardship (AMS). The aim of this evaluation was to explore the perceptions of healthcare workers from across the globe concerning various components of AMS training courses including technical content. **Methods:** This was an electronic cross-sectional survey sent to a convenience sample of 238 healthcare workers with expertise in infectious diseases (ID) and AMS from October to November 2025. Respondent demographic data, assessment of various components of AMS training courses using a 4-point Likert scale (Not Important, Somewhat Important, Important, Very Important), and ranking of AMS course content (1=most important to 9=least important) were collected. Descriptive analyses were used. **Results:** A total of 57 healthcare workers responded (24% response rate) including 32 physicians (58.2%) and 12 pharmacists (21.8%), mostly working in academic hospitals (73.2%) [Table 1]. The majority of respondents were aged 41-60 years (53.6%), and currently practicing in South America (31.5%), North America (24.5%), and South Asia (12.2%). The AMS course characteristics deemed very important by the majority of respondents included: free/low cost (69.5%), goals and aims described (66.1%), certificate of completion (64.3%), regularly updated materials addressing emerging issues in infectious diseases (63.6%), antimicrobial resistance specific to respondent’s country or community(62.5%), examples of implementation in the respondent’s specific geographical region (60.7%), no expiration on access to course content (58.2%), and appropriate time commitment (53.6%) [Table 2]. The course content topics identified as most important were AMS program fundamentals (45.5%) and application/implementation (34.5%), whereas ID basics (16.4%) was most frequently reported as least important. **Conclusion:** These data support the need for multi-faceted AMS training courses as most assessed components were identified as “important” or “very important” by this cohort of respondents. It also provides insights on what characteristics of a global AMS training course should be prioritized.

**Figure:**
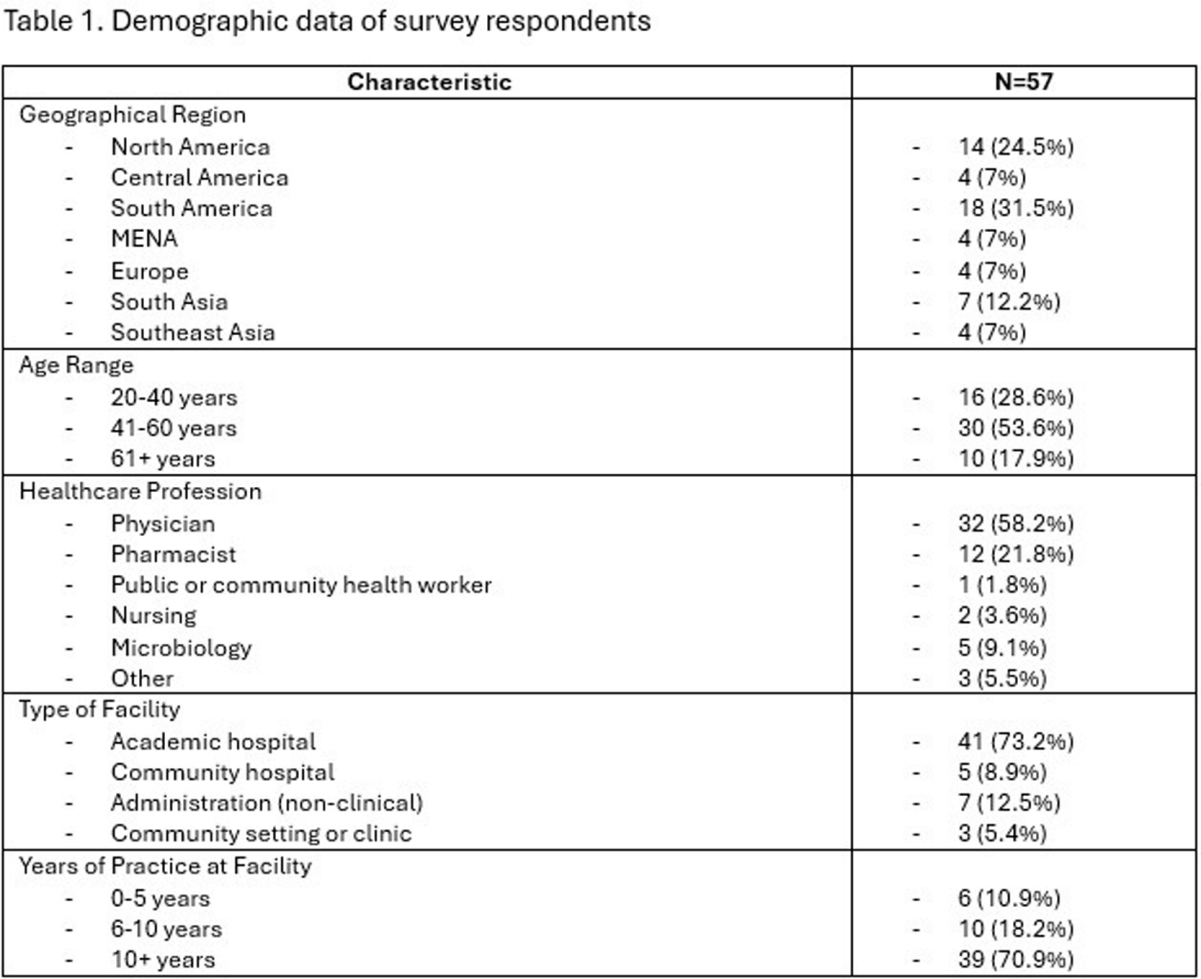

**Figure:**